# Association between a variation in the phosphodiesterase 4D gene and bone mineral density

**DOI:** 10.1186/1471-2350-6-9

**Published:** 2005-03-07

**Authors:** Richard H Reneland, Steven Mah, Stefan Kammerer, Carolyn R Hoyal, George Marnellos, Scott G Wilson, Philip N Sambrook, Tim D Spector, Matthew R Nelson, Andreas Braun

**Affiliations:** 1 Sequenom, Inc., San Diego, California, USA; 2 Department of Endocrinology & Diabetes, Sir Charles Gairdner Hospital, Perth, Australia; 3 Rheumatology Unit, Royal North Shore Hospital, NSW, Australia; 4 Twin Research & Genetic Epidemiology Unit, St Thomas Hospital, London, UK

## Abstract

**Background:**

Fragility fractures caused by osteoporosis are a major cause of morbidity and mortality in aging populations. Bone mineral density (BMD) is a useful surrogate marker for risk of fracture and is a highly heritable trait. The genetic variants underlying this genetic contribution are largely unknown.

**Methods:**

We performed a large-scale association study investigating more than 25,000 single nucleotide polymorphisms (SNPs) located within 16,000 genes. Allele frequencies were estimated in contrasting DNA pools from white females selected for low (<0.87 g/cm^2^, n = 319) and high (> 1.11 g/cm^2^, n = 321) BMD at the lumbar spine. Significant findings were verified in two additional sample collections.

**Results:**

Based on allele frequency differences between DNA pools and subsequent individual genotyping, one of the candidate loci indicated was the phosphodiesterase 4D (*PDE4D*) gene region on chromosome 5q12. We subsequently tested the marker SNP, rs1498608, in a second sample of 138 white females with low (<0.91 g/cm^2^) and 138 females with high (>1.04 g/cm^2^) lumbar spine BMD. Odds ratios were 1.5 (P = 0.035) in the original sample and 2.1 (P = 0.018) in the replication sample. Association fine mapping with 80 SNPs located within 50 kilobases of the marker SNP identified a 20 kilobase region of association containing exon 6 of *PDE4D*. In a second, family-based replication sample with a preponderance of females with low BMD, rs1498608 showed an opposite relationship with BMD at different sites (p = 0.00044-0.09). We also replicated the previously reported association of the Ser37Ala polymorphism in *BMP2*, known to interact biologically with PDE4D, with BMD.

**Conclusion:**

This study indicates that variants in the gene encoding PDE4D account for some of the genetic contribution to bone mineral density variation in humans. The contrasting results from different samples indicate that the effect may be context-dependent. PDE4 inhibitors have been shown to increase bone mass in normal and osteopenic mice, but up until now there have been no reports implicating any member of the *PDE4 *gene family in human osteoporosis.

## Background

The postmenopausal loss of bone mass and subsequent increased risk of low-energy (fragility) fractures is an important public health problem, especially in countries with a high proportion of elderly individuals. More than 1 million fragility fractures, primarily in postmenopausal women, occur each year in the US. The annual direct medical costs exceed US$10 billion [1]. Bone mineral density (BMD) measured with dual energy X-ray absorptiometry (DEXA) has been widely used to estimate the risk of fracture in epidemiological studies and to study treatment effects of antiresorptive agents in clinical trials. There are several well documented environmental and biological factors known to influence bone mineral density and the risk of fragility fractures including female gender, age, previous fragility fracture, low body weight, reduced lifetime exposure to estrogen, low calcium intake, physical inactivity, vitamin D deficiency, smoking, and excessive alcohol consumption [2-5]. There is also a strong genetic component to interindividual BMD variability, with heritability estimates ranging from 0.46 to 0.84 at different body sites [6-8]. Numerous candidate genes have been tested for association to BMD and fragility fractures. A polymorphism in a transcription factor-binding site of the collagen 1A1 (*COL1A1*) gene has shown one of the most consistent associations to osteoporosis, even if the association is generally weak for BMD and varies between populations [9-11]. Linkage studies have also been performed with the aim of locating genetic loci influencing BMD variation [12-19]. So far, the genes responsible for the resulting linkage peaks have not been identified. Recently, linkage of a compound osteoporosis phenotype was reported to chromosome 20p12. Subsequent positional cloning efforts implicated *BMP2*, the gene encoding for bone morphogenetic protein 2, as responsible for the linkage [20]. Nevertheless, the associations reported thus far that have been independently validated account for only a small portion of the genetic contribution to BMD and osteoporosis.

Studies that rely on direct association approaches based on linkage disequilibrium within populations are expected to have greater statistical power and be more feasible to implement than traditional linkage studies to identify common variations that influence common, complex traits such as osteoporosis [21]. Recently, there has been increasing interest in the use of whole-genome association methods to identify genes that are involved in complex trait variation. To date, however, few such large-scale studies have been reported. In an effort to identify genes and variants that influence risk of osteoporosis, we conducted a large-scale study using more than 25,000 single nucleotide polymorphisms (SNPs) located within approximately 16,000 genes in DNA pools of unrelated females at the extremes of the lumbar spine bone mineral density distribution. A number of intriguing associations identified in this study are currently being scrutinized in further detail. In this paper we report the most advanced of these, which is the association of a variation in *PDE4D*, the gene encoding cyclic AMP-dependent phosphodiesterase 4D, with lumbar spine BMD. PDE4D selective inhibitors have been shown to promote osteoblast differentiation in progenitor cells [22] and to increase bone mass by promoting bone formation in normal mice [23] but the gene has not until now been implicated in human bone metabolism.

## Methods

### Subjects

#### Discovery sample: unrelated females from UK twin collection

The population sample from which the discovery samples were chosen consisted of 5,436 female twins collected at the Twin Unit at St Thomas Hospital, London, England. They were selected without regard to health or trait. The volunteers had been recruited through advertisements and had undergone extensive investigation at the Twin Unit at St Thomas Hospital. Investigations included several questionnaires inquiring about present and past diseases, symptoms, family history, socio-economical factors, and medication. Subjects underwent an extensive clinical assessment including DEXA measurements of bone mineral density and anthropometric measurements [17,24]. All individuals with data on lumbar spine BMD were considered for inclusion. To exclude relatives, the individual with the most extreme BMD was kept in each twin pair. Individuals with diseases or medication known to influence BMD were excluded, as were individuals younger than 40 years because of the observed complex relationship between age and BMD. In addition, individuals with fractures were excluded from the high BMD group. BMD values were adjusted for age, weight, BMI, self reported leisure time physical activity, smoking, and menopausal status using an ordinary least squares model including second and third order polynomial terms for age and second order terms for weight and BMI. We included BMI and weight as covariates because both were independently associated with BMD in this sample. Based on the trade-off between group sizes and separation, target sizes of 350 were chosen, resulting in a separation of approximately 1.9 SD between groups. After assessment of DNA availability sufficient for such a large scale study, group sizes were reduced to 319 and 321 individuals in the high and low groups, respectively. Lumbar spine BMD T-scores were calculated with the females between 20 and 35 years of age as the reference population. Based on this, 32% of the women in the low BMD group had osteoporosis and an additional 58% had osteopenia according to WHO criteria. The characteristics of the selected individuals are reported in Table 1.

#### Replication sample: Australian twin collection

A twin sample from Royal North Shore Hospital, Sidney, Australia was collected similarly as the UK twin collection. 731 individuals including twin pairs and singletons with lumbar spine BMD assessments were available for genotyping (Table 3). Groups of unrelated subjects corresponding to the lower and upper quartiles of the age- and BMI-adjusted lumbar spine BMD distribution were defined similarly to the discovery sample. The characteristics of the selected individuals are reported in Table 1.

#### Replication sample: international multi-center sib-pair study

The second replication sample was a multi-center (Australia, UK, New Zealand, Belgium) study that collected sib pairs concordant and discordant for bone mineral density [17]. Probands (BMD Z-score <-1.5 at lumbar spine, femoral neck, or hip) were identified and their siblings were contacted and underwent DEXA measurements at the lumbar spine and hip. Participants had to be between 25–85 years of age. Exclusion criteria included steroid medication, hyperparathyroidism, immobility, amenorrhea, anorexia nervosa, and unstable thyroid disease. Nine hundred and eight individual samples were genotyped from 392 families. In the present analysis we included only females older than 40 years of age. In this sample the distribution of family sizes were 164 singletons, 248 families of 2, 34 families of 3, 7 families of 4, and 3 families of 5 members (Table 3). Lumbar spine BMD levels were adjusted by age and BMI as described for the Australian sample.

### Human subjects protection

All studies were approved by the appropriate research ethics committees. All participants gave their informed consent to participate in genetic studies before enrollment.

### Bone mineral density

Bone mineral density was estimated from the L1-L4 vertebrae, hip, and forearm using DEXA according to the user's manual for the Hologic QDR 4500W, (Hologic, Waltham, Massachusetts, United States) at all collection sites.

### SNP markers and genotyping

A set of 25,494 SNP markers was selected from a collection of 125,799 experimentally validated polymorphic variations [25]. This set was limited to SNPs located within gene coding regions, minor allele frequencies greater than 0.02 (95% have frequencies greater than 0.1), and a target inter-marker spacing of 40 kb. SNP annotation is based on NCBI dbSNP database, refSNP, build 118. Genomic annotation is based on NCBI Genome Build 34. Gene annotation is based on LocusLink genes for which NCBI was providing positions on the Mapview FTP site.

For pooled DNA assays, 25 ng of case and control DNA pools was used for amplification at each site. All PCR and MassEXTEND™ reactions were conducted using standard conditions [26]. Relative allele frequency estimates were derived from area under the peak calculations of mass spectrometry measurements from four analyte aliquots as described elsewhere [26]. For individual genotyping, the same procedure was applied except only 2.5 ng DNA was used and only one mass spectrometry measurement was taken.

Primers used to genotype rs1498608 were ATAACCTCGGGGTCCAGAAA (forward PCR primer), GAATCCCTGTTCATTCCTTG (reverse PCR primer) and CCCTAAAAACTGTTCCAGGTA (extension primer). The primers used to genotype the Ser37Ala polymorphism in *BMP2 *were AGCTGGGCCGCAGGAAGTTCG (forward PCR primer), TCGTCAGAGGGCTGGGATGAG (reverse PCR primer) and TGAGGGGCGGCCCGACG (extension primer).

### Statistical analysis

Tests of association between adjusted lumbar spine BMD group and each SNP using pooled DNA were carried out in a similar fashion as explained elsewhere [27]. Briefly, the test statistic is based on the difference in allele frequencies between the two groups divided by the known sources of variation in each allele frequency estimate, including sampling and pool-specific measurement variation. Sources of measurement variation incorporated in the test statistic are pool formation, PCR/mass extension, and chip measurement. When three or more replicate measurements of a SNP were available within a model level, the corresponding variance component was estimated from the data. Otherwise, the following historical laboratory averages were used: pool formation = 5.0 × 10^-5^, PCR/mass extension = 1.7 × 10^-4^, and chip measurement = 1.0 × 10^-4^.

Tests of association using individual genotypes were carried out using a chi-square test of heterogeneity to compare allele frequencies, and Fisher's exact test to compare genotype frequencies (due to low frequency contingency table cells). Confidence intervals and P-values for odds ratios were derived using Fisher's exact test. When one or more cell counts were zero, non-infinite odds ratios were estimated by adding 0.5 to each cell [28]. In the samples that included a combination of singletons, sib pairs, and occasionally additional relatives, we estimated the relationship between genotypes and phenotypes using the generalized estimating equations (GEE) approach with a Gaussian link by clustering on family using an exchangeable correlation matrix [29]. Hypothesis testing was carried out with a Wald test statistic. The geepack implementation of GEE in the R statistical software platform was used [30]. No attempt was made to correct P-values for multiple testing. Rather, P-values are provided to compare the relative strength of association. P-values less than 0.05 are referred to as statistically significant.

## Results

### Initial genome scan in UK sample

**Figure 1:**
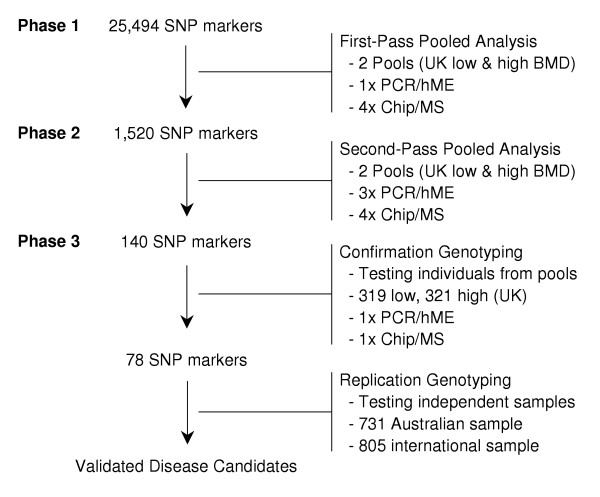
Overview of DNA pool-based large-scale association strategy. Phases 1 and 2 are conducted using DNA pools yielding allele frequencies, all subsequent steps involve genotyping of individual samples. hME, homogeneous MassEXTEND; MS, mass spectrometry. See text for more details.

We carried out a genome-wide association study using 25,494 SNPs located within 10 kb of 15,995 LocusLink annotated genes. An overview of the investigative process is shown in Figure 1. The basic design was a two-group study in subjects from the tails of the adjusted lumbar spine BMD distribution. We selected lumbar spine BMD as the phenotype to create the contrasting groups because it had a high estimate of heritability in our twin sample (h^2 ^= 0.82) [7]. The selected low and high BMD groups consisted of 319 and 321 individuals, respectively. The adjusted BMD range was 0.56–0.87 g/cm^2 ^in the low BMD group and 1.11–1.60 g/cm^2 ^in the high BMD group, corresponding to the upper and lower 22^nd ^percentiles. Other characteristics of the samples are described in Table 1. To facilitate the screening of such a large number of SNPs, we employed a high-throughput approach using DNA pools, chip-based mass spectrometry [26,31-33], and a three-phase SNP selection strategy (Figure 1). In the first phase, we performed a single PCR and primer extension reaction for each SNP on two DNA pools consisting of equimolar amounts of DNA from each individual in the low BMD group and high BMD group, respectively. Relative allele frequencies obtained from four mass spectrometry measurements of the extension products were compared between pools. In the second phase, the 1,520 SNPs (~6%) with the most statistically significant associations (nominal P-values < 0.05) were re-measured in triplicate on each DNA pool. In the third phase, we genotyped the 140 most significant SNPs (9%) from step two (nominal P-values < 0.02) on all individuals comprising the pools. Based on the genotype results, 78 SNPs were confirmed to have statistically significant allele frequency differences between cases and controls (P < 0.05). The liberal criteria for selecting SNPs from each phase represent a practical trade-off between following up false positive versus overlooking false negative associations. We chose to follow up as many SNPs from each phase as seemed reasonable.

One of the associations was found with rs1498608, an A/T polymorphism within intron 5 of *PDE4D *on chromosome 5q12. Allele frequencies for the T allele based on genotyping were 0.91 in the low lumbar spine BMD pool and 0.88 in the high BMD pool (OR = 1.5, P = 0.035). Complete genotype counts and summary statistics are reported in Table 2. Observed genotype frequencies were consistent with expected frequencies under Hardy-Weinberg equilibrium within each study collection. Menopausal status did not have a significant influence on the effect (P = 0.87).

Genome-wide studies using tens of thousands of SNPs and liberal statistical selection criteria are expected to yield a high proportion of false positive associations. To distinguish the true genetic effects from among the false positives, the 78 selected SNPs were genotyped in a second twin sample from Australia.

### Replication in Australian sample

The Australian replication sample, a combination of female and male twin pairs and singletons, was analyzed in two ways. First, to create a design and carry out an analysis comparable to the discovery sample, unrelated individuals were selected from the lower and upper quartiles of the sex-specific adjusted lumbar spine BMD distribution (Table 1). A similar effect was observed for the marker SNP rs1498608 in females (OR = 2.14, P = 0.018) and males (OR = 1.55, P = 0.35) as in the original UK collection (Table 2). The second method of analysis utilized generalized estimating equation (GEE) models to take into account all of the available genotype information by carrying out a regression-type analysis while accounting for familial covariance. The characteristics of this sample are reported in Table 3. The regression of marker genotypes on adjusted lumbar spine BMD, with sex included as a covariate, found the AA genotype to be associated with significantly higher levels than the AT (β = 7.8 g/cm^2^, P = 0.049) or TT (β = 8.0 g/cm^2^, P = 0.037) genotypes, thus confirming the results observed in the unrelated tails of this sample. Similar GEE analyses carried out for femoral neck and hip BMD were not statistically significant.

### Replication in international multi-center family study

Being a sample of mostly affected sib pairs, this sample was unsuitable for formation of groups with contrasting BMD because of the preponderance of individuals with low BMD (Table 3). Therefore, we restricted the analysis to using a generalized estimating equation, regressing marker genotypes on BMD values. Surprisingly, the estimates in this sample were opposite to that in the Australian sample, as the AA genotype was associated with lower adjusted lumbar spine BMD values than both the AT (β = -5.3 g/cm^2^, P = 0.11) and the TT (β = -5.4 g/cm^2^, P = 0.09) genotypes. Using Z-scores at the femoral neck (P = 0.0007 and 0.0004), total hip (P = 0.003 and 0.007), and lumbar spine (P = 0.03 and 0.02) as dependent variables confirmed this pattern of association. In all cases the AT and TT genotypes had very similar point estimates.

### Association fine mapping

**Figure 2:**
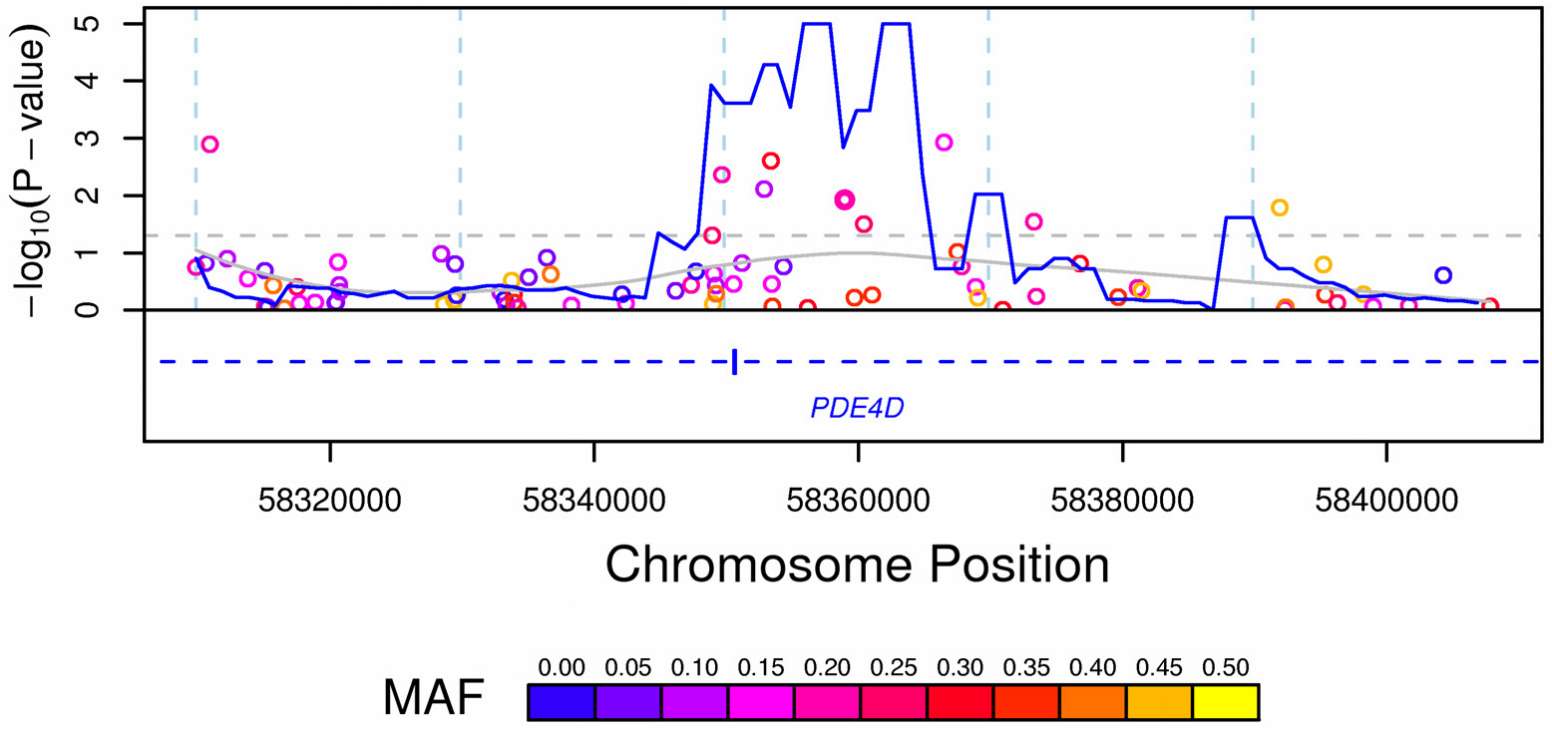
Association fine mapping of *PDE4D *region. Eighty public domain SNPs in a 100-kb window around the initial marker SNP (indicated as a bold symbol at the center of the plot) were compared between the UK low and high BMD pools. Ten of 80 SNPs were significant at P = 0.05 (horizontal dashed line). The *x*-axis corresponds to the chromosomal position of each SNP, the *y*-axis to the test P-values (--log_10 _scale). The continuous dark line presents the results of a goodness-of-fit test for an excess of significant associations (at a 5% significance level) in a 10-kb sliding window assessed at 1-kb increments. The continuous light gray line is the result of a nonlinear smoothing function showing a weighted average of the P-values across the region. The color of each point corresponds to the minor allele frequency of each SNP in the control sample (see legend below graph). Vertical dashed lines are placed every 20 kb. The LocusLink gene annotation for NCBI genome build 34 shows the location of exon six. All investigated SNPs are located within introns.

In order to better define the extent of the region of association and possibly identify other SNPs more strongly associated with BMD, we performed DNA pool based association fine mapping in the UK sample using 80 publicly available intronic SNPs in the 100 kb region surrounding the incident SNP (Figure 2). This analysis identified a 20 kb region of association encompassing exon 6 of *PDE4D*.

### Replication of *BMP2 *association

As described in the discussion below, PDE4D inhibition is known to influence BMP2-induced alkaline phosphatase activity in osteoblast precursor cells. Recently, variation in the gene encoding BMP2 was found to be associated with osteoporosis in a study employing whole genome linkage and subsequent positional cloning [20]. Since we were unaware of any published independent attempts to replicate this finding, we genotyped the Ser37Ala polymorphism in our UK and international samples. In the UK sample, the allele frequency of the rare allele was 2.2% in the low BMD group and 1.4% in the high BMD group, with an odds ratio of 1.6 (P = 0.28). We tested for, but were unable to detect, an interaction between the Ser37Ala polymorphism and rs1498608 on the association with lumbar spine BMD. In the international sample we performed an allele based general estimating equation to estimate the effect of the rare allele on BMD in that sample. The allele frequency of Ala37 in this sample, mainly selected for low BMD, was 1.9%. The effect of the Ala allele was estimated to decrease the adjusted lumbar spine BMD by 0.06 g/cm^2 ^(P = 0.0029). There were no homozygous Ala individuals in this sample.

## Discussion

In an association study using SNPs in nearly 16,000 genes we obtained evidence that variation in the SNP rs1498608 located within *PDE4D *is associated with low bone mineral density at the lumbar spine in females. *PDE4D *encodes cyclic AMP-dependent phosphodiesterase 4D. Phosphodiesterases are a superfamily of enzymes involved in degradation of cyclic adenosine monophosphate (cAMP) and cyclic guanosine monophosphate (cGMP) [34,35]. cAMP and cGMP are important second messengers participating in the response of various cells to hormones. In osteoblasts, cAMP produced in response to parathyroid hormone or prostaglandins regulates osteoblastic differentiation [36-39], which leads to increases in cancellous bone volume as indicated by experiments in animal models [40-45]. The intracellular level of cAMP is regulated by G protein-coupled adenylyl cyclase [46], and degradation is mediated by the phosphodiesterases. The phosphodiesterase superfamily consists of seven families, PDE1-7, distinguished by substrate specificity, chromatographic behaviour during purification, and affinity for biochemical activators and inhibitors. Of these, the PDE4 family is specific for cAMP and is selectively inhibited by rolipram. Four PDE4 genes, 4A, 4B, 4C, and 4D, have been cloned from rat and humans, all of which are predicted to have multiple protein products due to alternate spicing of RNAs. PDE4 inhibitors have been shown to increase bone formation in normal mice [23] and to ameliorate loss of bone mass in animal models of osteopenia [47,48]. PDE4A and PDE4D are expressed in two common mouse osteoblastic cell lines, ST2 and MC3T3-E1, that represent different stages in the osteoblast differentiation pathway [22]. PDE4 inhibition with rolipram increased BMP2-induced alkaline phosphatase activity, a marker of early osteoblast differentiation in ST2 cells. Furthermore, rolipram increased the expression of alkalic phosphatase, osteopontin, collagen type I and osteocalcin in the same osteoblast precursor cells [22]. In spite of these experimental data, we are not aware of any published attempts to investigate the role of *PDE4 *genes in human osteoporosis. However, variation in *PDE4D *was recently reported to be associated with the risk of ischemic stroke [49]. Given the central role of PDE4 in second messenger signalling, it is quite conceivable that *PDE4D *variants may have effects on the risk for different common diseases. There are other examples of genes having such pleiotropic effects, the most notable being *APOE *in hyperlipidemia and Alzheimer's disease [50,51]. It should also be noted that Gretarsdottir et al found that the *PDE4D *association with stroke was strongest for a region in the recently extended 5' end of the gene, which is close to 1,000 kb upstream of rs1498608 [49]. Assuming a contribution of *PDE4D *to the risk of osteoporosis as well as stroke, it is possible that different domains are involved in the different diseases.

Given the interaction between BMP2 and PDE4 for the inhibition on osteoblastic differentiation in vitro, it is interesting to note that variants in the gene encoding for BMP2 have also been found to increase risk of osteoporosis in humans [20]. In the current study, we replicated the association between the Ser37Ala variant in *BMP2 *and measures of osteoporosis in an international family-based sample ascertained via low BMD probands. Although not statistically significant, this finding was supported by the results in the discovery sample of unrelated high and low spine BMD subjects. The allele frequency in the low BMD group was 2.2% and in the high group 1.4%, with an odds ratio of 1.6 (P = 0.28). The rare allele was less common in our low BMD group than the low spine BMD group (3%) in the Icelandic sample. However, our allele frequencies in the low and high BMD groups and the resulting OR corresponded well with the figures in the Danish sample (1.8% vs 1.0%, RR = 1.8) reported in the same paper [20]. We found no evidence for statistical interaction between the variations in *BMP2 *and *PDE4D *in either sample. However, given the low minor allele frequencies of each SNP, there was very little power to test for interaction effects.

The starting point of the present study was a large-scale association study of more than 25,000 SNPs located in 16,000 genes. After a stepwise selection process an association between an intronic SNP in *PDE4D *and lumbar spine bone mineral density was detected, providing the first evidence that a variant of this gene could contribute to the risk of osteoporosis in humans. The effect was similar in size in premenopausal and postmenopausal women, indicating that the effect would be on the attainment of peak bone mass rather than the rate of decrease in BMD after menopause. The lack of a detectable interaction with female sex hormones is supported by having observed a similar genetic effect in the small sample of males in our study. The genetic contribution to peak bone mass is possibly bigger and definitely better documented than the as yet unproven genetic influence on postmenopausal bone loss [52], and it is possible that *PDE4D *could contribute to this effect, especially in light of the documented anabolic effect on bone by PDE4 inhibitors.

An association with an intronic SNP provides little evidence for a change in amount or function of the protein that could explain the association. None of the 80 SNPs investigated as part of the association fine mapping were non-synonymous coding changes, which is consistent with the extraordinary lack of variation that others have reported for all PDE classes [53] and PDE4D in particular [49]. This makes it unlikely (but still possible) that the observed association would be due to a non-synonymous and disruptive single-base coding change in linkage disequilibrium with our marker SNP. Therefore it is more likely that the effect is mediated by a change in RNA splicing or expression.

Given the functional similarity between different PDE4 enzymes, we went back and scrutinized our data for associations with SNPs in the other PDE4 genes that may have been overlooked during the first stage of the scan. The only SNP in *PDE4B *in our assay set, rs1318475, was taken through to the second stage (Figure 1) where it was estimated to have an OR of 0.78 (P = 0.041), but failed the criteria to be taken forward to the genotyping stage. Similarly, a SNP roughly 18 kb downstream of *PDE4C*, rs874628, was also taken forward to the second stage where it displayed an OR of 1.3 (P = 0.08). These results suggest that further investigation into possible associations between variants in all PDE4 genes and bone mineral density may be justified.

The route by which these genetic associations were arrived at and the potential for spurious association must be considered. Recent published work has brought light to the need for proper validation to verify genetic findings for complex traits [54-56]. In the current study, the initial association found between the *PDE4D *marker and bone mineral density was one result from over 25,000 hypothesis tests. A conservative Bonferroni adjustment to yield an experiment-wide type I error rate of 0.05 would demand a test-wise p-value on the order of 10^-6^. Given the modest sample size, only common variations with relatively large effects (OR > 2) would reach such significance levels. Instead, we chose to be more mindful of the role of type II error rates and apply a more liberal set of criteria in the initial phases of the study and verify true genetic effects by independent replication. The analysis of 78 selected markers in the Australian replication sample resulted in multiple associations of continuing interest, with rs1498608 displaying one of the strongest associations. A one-sided test of association comparing the results in the discovery and replication samples yields a p-value of 0.0074. This would not be considered significant on an experiment-wide level after Bonferroni adjustment, which would require a p-value on the order of 0.0006 or lower. The analysis in the international replication sample produced contradictory data in that the A allele, which in the first two samples was associated with increased lumbar spine bone mineral density, was associated with decreased BMD at all tested sites. The pattern of association evident from the first two samples, with AT and TT genotypes having very similar point estimates, was preserved in this sample, even in the face of the reverse direction of association. The highly statistically significant association between rs1498608 and femoral neck and hip BMD in this third sample and the consistency in the pattern of association would be unexpected from a spurious result. A possible explanation for the contradictory results could be the fact that the international sample consists mostly of individuals with low BMD since the probands all have a BMD Z-score < -1.5, and most of the siblings also have low BMD. It is possible that within such a selected sample the relationship between rs1498608 and BMD could be altered due to interactions with other genetic or environmental factors.

The well-documented anabolic effect on bone by PDE4 inhibitors lends indirect support for the association reported here, and it would seem that the possible role of *PDE4D *variants in the genetic contribution to BMD in humans merits further investigation.

## Conclusion

The result of the present large scale association study together with data from previously published animal models suggest that genetic variation in the gene encoding PDE4D contributes to the variation in lumbar spine BMD in humans.

## Competing interests

The authors of this manuscript affiliated with Sequenom, Inc. (RR, SM, CH, GM, SK, MN, AB) declare competing financial interests, which may include current or prior receipt of salary and/or stock ownership, as Sequenom, Inc. may be affected financially by the publication of this manuscript.

## Authors' contributions

RR drafted the manuscript and participated in study design and data analysis. SM was study project leader and participated in data analysis. SK supervised the operational aspects of the study, and was responsible for the development of the SNP marker assay set. CH and GM participated in the development of the SNP marker assay set. SW was project manager for the international multicenter study. PS was principal investigator for the Australian Twin collection. TS was principal investigator for the UK twin and international collections. MN participated in study design, performed the statistical analyses and helped draft the manuscript. AB had the overall scientific responsibility for the study.

## Pre-publication history

The pre-publication history for this paper can be accessed here:



## Figures and Tables

**Table 1:** Characteristics of subjects in the groups selected for low and high lumbar spine bone mineral density.

	Discovery (UK)		Replication (AUS)		Replication (AUS)	
	Females		Females		Males	
	
Trait	Low BMD (N = 319)	High BMD (N = 321)	Low BMD (N = 138)	High BMD (N = 138)	Low BMD (N = 34)	High BMD (N = 34)
Age (years)	48/52/60	48/53/59	44/52/61	45/53/61	48/56/65	48/54/62

Menopausal status						
Pre	29% (76)	31% (84)	41% (45)	36% (41)	N/A	N/A
Post	71% (184)	69% (190)	59% (66)	64% (73)		
Smoking						
Never	56% (177)	53% (168)	78% (93)	67% (93)	41% (14)	62% (21)
Former	26% (82)	29% (93)	22% (13)	20% (28)	47% (16)	21% (7)
Current	18% (56)	18% (57)	10% (13)	13% (17)	12% (4)	18% (6)
Weight (kg)	58/66/74	58/64/72	56/64/74	58/67/76	72/80/87	74/80/90
BMI	23/25/28	22/25/27	22/25/27	23/26/29	24/27/28	24/26/29
BMD spine (g/cm^2^)	0.74/0.80/0.87	1.12/1.19/1.26	0.77/0.87/0.96	0.98/1.10/1.17	0.88/0.92/1.03	1.00/1.14/1.21
Adjusted BMD spine (g/cm^2^)	0.77/0.81/0.84	1.14/1.18/1.23	0.80/0.84/0.87	1.08/1.13/1.19	0.86/0.90/0.93	1.15/1.17/1.25
BMD hip (g/cm^2^)	0.75/0.82/0.89	0.95/1.02/1.10	0.79/0.87/0.95	0.91/0.97/1.06	0.96/1.03/1.11	1.02/1.08/1.20
BMD femoral neck (g/cm^2^)	0.63/0.70/0.77	0.82/0.89/0.98	0.64/0.73/0.81	0.75/0.84/0.91	0.77/0.83/0.92	0.80/0.88/0.99
BMD forearm (g/cm^2^)	0.48/0.52/0.56	0.55/0.59/0.61	0.49/0.53/0.57	0.53/0.56/0.59	0.59/0.62/0.68	0.64/0.67/0.73

Categorical variables are reported as frequency percent (count). Quantitative variables are reported as 1^st ^quartile/median/3^rd ^quartile.

**Table 2:** Allele and genotype frequencies of rs1498608 in high and low lumbar spine BMD groups.

Sample	Allele/Genotype	Low N (Rel. Freq.)	High N (Rel. Freq.)	OR (95% C.I.)	P-value
Discovery (UK)					
Female	A	52 (0.086)	77 (0.123)		0.035^a^
	T	550 (0.914)	547 (0.877)	1.5 (1.0, 2.2)	
	
	AA	1 (0.003)	9 (0.029)		0.026^b^
	AT	50 (0.166)	59 (0.189)	7.6 (0.99, 340)	0.042^c^
	TT	250 (0.831)	244 (0.782)	9.2 (1.3, 410)	0.020^c^

Replication (AUS)					
Female	A	15 (0.054)	30 (0.109)		0.018^a^
	T	261 (0.946)	244 (0.891)	2.1 (1.1, 4.4)	
	
	AA	0 (0.000)	3 (0.022)		0.048^b^
	AT	15 (0.109)	24 (0.175)	4.4 (0.23, ∞)	0.541^c^
	TT	123 (0.891)	110 (0.803)	7.8 (0.45, ∞)	0.108^c^
	
					
Male	A	9 (0.132)	13 (0.191)		0.352^a^
	T	59 (0.868)	55 (0.809)	1.6 (0.56, 4.5)	
	
	AA	1 (0.029)	3 (0.088)		0.754^b^
	AT	7 (0.206)	7 (0.206)	3.0 (0.17, 180)	0.588^c^
	TT	26 (0.765)	24 (0.706)	3.3 (0.24, 180)	0.610^c^

Abbreviations: Rel. Freq., relative frequency; OR, odds ratio; C.I., confidence interval. Odds ratios for the AT and TT genotypes are contrasted against the AA genotype. ^a^Chi-square test of heterogeneity comparing allele frequencies between high versus low. ^b^Fisher's exact test comparing three genotype frequencies between high versus low. ^c^Fisher's exact test comparing genotype frequencies in current table row and AA genotype between high versus low.

**Table 3:** Characteristics of complete genotyped replication samples.

	Australian Twin Replication		International Replication
Trait	Females (N = 590)	Males (N = 141)	Females (N = 805)

Age (years)	40/52/61	47/54/64	52/59/67

Twin status			
Singletons	42% (246)	48% (67)	N/A
Dizygotic pairs	58% (72)	52% (37)	N/A
Smoking status			
Never	63% (368)	55% (78)	52% (317)
Former	24% (141)	30% (42)	26% (157)
Current	13% (77)	15% (21)	22% (130)
Weight (kg)	58/64/74	73/81/91	55/60/69
BMI	22/25/28	25/27/29	22/24/27
BMD spine (g/cm^2^)	0.89/0.99/1.09	0.92/1.03/1.13	0.71/0.80/0.94
Adjusted BMD spine (g/cm^2^)	0.89/0.99/1.06	0.93/1.02/1.12	0.74/0.81/0.92
BMD hip (g/cm^2^)	0.85/0.93/1.02	0.96/1.05/1.14	0.68/0.77/0.87
BMD femoral neck (g/cm^2^)	0.71/0.79/0.88	0.78/0.86/0.95	0.57/0.65/0.73
BMD forearm (g/cm^2^)	0.51/0.55/0.59	0.61/0.66/0.69	N/A

Categorical variables are reported as frequency percent (count). Quantitative variables are reported as 1^st ^quartile / median / 3^rd ^quartile.

## References

[B1] Ray NF, Chan JK, Thamer M, Melton LJ (1997). Medical expenditures for the treatment of osteoporotic fractures in the United States in 1995: report from the National Osteoporosis Foundation. J Bone Miner Res.

[B2] Cummings SR, Nevitt MC, Browner WS, Stone K, Fox KM, Ensrud KE, Cauley J, Black D, Vogt TM (1995). Risk factors for hip fracture in white women. Study of Osteoporotic Fractures Research Group. N Engl J Med.

[B3] Espallargues M, Sampietro-Colom L, Estrada MD, Sola M, del Rio L, Setoain J, Granados A (2001). Identifying bone-mass-related risk factors for fracture to guide bone densitometry measurements: a systematic review of the literature. Osteoporos Int.

[B4] Kanis JA (2002). Diagnosis of osteoporosis and assessment of fracture risk. Lancet.

[B5] Jordan KM, Cooper C (2002). Epidemiology of osteoporosis. Best Pract Res Clin Rheumatol.

[B6] Arden NK, Baker J, Hogg C, Baan K, Spector TD (1996). The heritability of bone mineral density, ultrasound of the calcaneus and hip axis length: a study of postmenopausal twins. J Bone Miner Res.

[B7] Hunter DJ, de Lange M, Andrew T, Snieder H, MacGregor AJ, Spector TD (2001). Genetic variation in bone mineral density and calcaneal ultrasound: a study of the influence of menopause using female twins. Osteoporos Int.

[B8] Pocock NA, Eisman JA, Hopper JL, Yeates MG, Sambrook PN, Eberl S (1987). Genetic determinants of bone mass in adults. A twin study. J Clin Invest.

[B9] Efstathiadou Z, Kranas V, Ioannidis JP, Georgiou I, Tsatsoulis A (2001). The Sp1 COLIA1 gene polymorphism, and not vitamin D receptor or estrogen receptor gene polymorphisms, determines bone mineral density in postmenopausal Greek women. Osteoporos Int.

[B10] Efstathiadou Z, Tsatsoulis A, Ioannidis JP (2001). Association of collagen Ialpha 1 Sp1 polymorphism with the risk of prevalent fractures: a meta-analysis. J Bone Miner Res.

[B11] Mann V, Hobson EE, Li B, Stewart TL, Grant SF, Robins SP, Aspden RM, Ralston SH (2001). A COL1A1 Sp1 binding site polymorphism predisposes to osteoporotic fracture by affecting bone density and quality. J Clin Invest.

[B12] Devoto M, Shimoya K, Caminis J, Ott J, Tenenhouse A, Whyte MP, Sereda L, Hall S, Considine E, Williams CJ, Tromp G, Kuivaniemi H, Ala-Kokko L, Prockop DJ, Spotila LD (1998). First-stage autosomal genome screen in extended pedigrees suggests genes predisposing to low bone mineral density on chromosomes 1p, 2p and 4q. Eur J Hum Genet.

[B13] Niu T, Chen C, Cordell H, Yang J, Wang B, Wang Z, Fang Z, Schork NJ, Rosen CJ, Xu X (1999). A genome-wide scan for loci linked to forearm bone mineral density. Hum Genet.

[B14] Koller DL, Econs MJ, Morin PA, Christian JC, Hui SL, Parry P, Curran ME, Rodriguez LA, Conneally PM, Joslyn G, Peacock M, Johnston CC, Foroud T (2000). Genome screen for QTLs contributing to normal variation in bone mineral density and osteoporosis. J Clin Endocrinol Metab.

[B15] Deng HW, Xu FH, Liu YZ, Shen H, Deng H, Huang QY, Liu YJ, Conway T, Li JL, Davies KM, Recker RR (2002). A whole-genome linkage scan suggests several genomic regions potentially containing QTLs underlying the variation of stature. Am J Med Genet.

[B16] Karasik D, Myers RH, Cupples LA, Hannan MT, Gagnon DR, Herbert A, Kiel DP (2002). Genome screen for quantitative trait loci contributing to normal variation in bone mineral density: the Framingham Study. J Bone Miner Res.

[B17] Wilson SG, Reed PW, Bansal A, Chiano M, Lindersson M, Langdown M, Prince RL, Thompson D, Thompson E, Bailey M, Kleyn PW, Sambrook P, Shi MM, Spector TD (2003). Comparison of genome screens for two independent cohorts provides replication of suggestive linkage of bone mineral density to 3p21 and 1p36. Am J Hum Genet.

[B18] Econs MJ, Koller DL, Hui SL, Fishburn T, Conneally PM, Johnston CCJ, Peacock M, Foroud TM (2004). Confirmation of linkage to chromosome 1q for peak vertebral bone mineral density in premenopausal white women. Am J Hum Genet.

[B19] Peacock M, Koller DL, Hui S, Johnston CC, Foroud T, Econs MJ (2004). Peak bone mineral density at the hip is linked to chromosomes 14q and 15q. Osteoporos Int.

[B20] Styrkarsdottir U, Cazier JB, Kong A, Rolfsson O, Larsen H, Bjarnadottir E, Johannsdottir VD, Sigurdardottir MS, Bagger Y, Christiansen C, Reynisdottir I, Grant SF, Jonasson K, Frigge ML, Gulcher JR, Sigurdsson G, Stefansson K (2003). Linkage of Osteoporosis to Chromosome 20p12 and Association to BMP2. PLoS Biol.

[B21] Risch NJ (2000). Searching for genetic determinants in the new millennium. Nature.

[B22] Wakabayashi S, Tsutsumimoto T, Kawasaki S, Kinoshita T, Horiuchi H, Takaoka K (2002). Involvement of phosphodiesterase isozymes in osteoblastic differentiation. J Bone Miner Res.

[B23] Kinoshita T, Kobayashi S, Ebara S, Yoshimura Y, Horiuchi H, Tsutsumimoto T, Wakabayashi S, Takaoka K (2000). Phosphodiesterase inhibitors, pentoxifylline and rolipram, increase bone mass mainly by promoting bone formation in normal mice. Bone.

[B24] Andrew T, Mak YT, Reed P, MacGregor AJ, Spector TD (2002). Linkage and association for bone mineral density and heel ultrasound measurements with a simple tandem repeat polymorphism near the osteocalcin gene in female dizygotic twins. Osteoporos Int.

[B25] Nelson MR, Marnellos G, Kammerer S, Hoyal CR, Shi MM, Cantor CR, Braun A (2004). Large-scale validation of single nucleotide polymorphisms in gene regions. Genome Res.

[B26] Bansal A, van den Boom D, Kammerer S, Honisch C, Adam G, Cantor CR, Kleyn P, Braun A (2002). Association testing by DNA pooling: an effective initial screen. Proc Natl Acad Sci U S A.

[B27] Barratt BJ, Payne F, Rance HE, Nutland S, Todd JA, Clayton DG (2002). Identification of the sources of error in allele frequency estimations from pooled DNA indicates an optimal experimental design. Ann Hum Genet.

[B28] Agresti A (2002). Categorical data analysis.

[B29] Slager SL, Schaid DJ, Wang L, Thibodeau SN (2003). Candidate-gene association studies with pedigree data: controlling for environmental covariates. Genet Epidemiol.

[B30] Yan J, Fine J (2004). Estimating equations for association structures. Stat Med.

[B31] Kammerer S, Burns-Hamuro LL, Ma Y, Hamon SC, Canaves JM, Shi MM, Nelson MR, Sing CF, Cantor CR, Taylor SS, Braun A (2003). Amino acid variant in the kinase binding domain of dual-specific A kinase-anchoring protein 2: a disease susceptibility polymorphism. Proc Natl Acad Sci U S A.

[B32] Buetow KH, Edmonson M, MacDonald R, Clifford R, Yip P, Kelley J, Little DP, Strausberg R, Koester H, Cantor CR, Braun A (2001). High-throughput development and characterization of a genomewide collection of gene-based single nucleotide polymorphism markers by chip-based matrix-assisted laser desorption/ionization time-of-flight mass spectrometry. Proc Natl Acad Sci U S A.

[B33] Mohlke KL, Erdos MR, Scott LJ, Fingerlin TE, Jackson AU, Silander K, Hollstein P, Boehnke M, Collins FS (2002). High-throughput screening for evidence of association by using mass spectrometry genotyping on DNA pools. Proc Natl Acad Sci U S A.

[B34] Manganiello VC, Murata T, Taira M, Belfrage P, Degerman E (1995). Diversity in cyclic nucleotide phosphodiesterase isoenzyme families. Arch Biochem Biophys.

[B35] Beavo JA (1995). Cyclic nucleotide phosphodiesterases: functional implications of multiple isoforms. Physiol Rev.

[B36] Farndale RW, Sandy JR, Atkinson SJ, Pennington SR, Meghji S, Meikle MC (1988). Parathyroid hormone and prostaglandin E2 stimulate both inositol phosphates and cyclic AMP accumulation in mouse osteoblast cultures. Biochem J.

[B37] Kumegawa M, Ikeda E, Tanaka S, Haneji T, Yora T, Sakagishi Y, Minami N, Hiramatsu M (1984). The effects of prostaglandin E2, parathyroid hormone, 1,25 dihydroxycholecalciferol, and cyclic nucleotide analogs on alkaline phosphatase activity in osteoblastic cells. Calcif Tissue Int.

[B38] Ishizuya T, Yokose S, Hori M, Noda T, Suda T, Yoshiki S, Yamaguchi A (1997). Parathyroid hormone exerts disparate effects on osteoblast differentiation depending on exposure time in rat osteoblastic cells. J Clin Invest.

[B39] Partridge NC, Bloch SR, Pearman AT (1994). Signal transduction pathways mediating parathyroid hormone regulation of osteoblastic gene expression. J Cell Biochem.

[B40] Jee WS, Ueno K, Deng YP, Woodbury DM (1985). The effects of prostaglandin E2 in growing rats: increased metaphyseal hard tissue and cortico-endosteal bone formation. Calcif Tissue Int.

[B41] Jee WS, Ueno K, Kimmel DB, Woodbury DM, Price P, Woodbury LA (1987). The role of bone cells in increasing metaphyseal hard tissue in rapidly growing rats treated with prostaglandin E2. Bone.

[B42] High WB (1987). Effects of orally administered prostaglandin E-2 on cortical bone turnover in adult dogs: a histomorphometric study. Bone.

[B43] Whitfield JF, Morley P (1995). Small bone-building fragments of parathyroid hormone: new therapeutic agents for osteoporosis. Trends Pharmacol Sci.

[B44] Reeve J (1996). PTH: a future role in the management of osteoporosis?. J Bone Miner Res.

[B45] Finkelstein JS, Klibanski A, Schaefer EH, Hornstein MD, Schiff I, Neer RM (1994). Parathyroid hormone for the prevention of bone loss induced by estrogen deficiency. N Engl J Med.

[B46] Casperson GF, Bourne HR (1987). Biochemical and molecular genetic analysis of hormone-sensitive adenylyl cyclase. Annu Rev Pharmacol Toxicol.

[B47] Miyamoto K, Waki Y, Horita T, Kasugai S, Ohya K (1997). Reduction of bone loss by denbufylline, an inhibitor of phosphodiesterase 4. Biochem Pharmacol.

[B48] Waki Y, Horita T, Miyamoto K, Ohya K, Kasugai S (1999). Effects of XT-44, a phosphodiesterase 4 inhibitor, in osteoblastgenesis and osteoclastgenesis in culture and its therapeutic effects in rat osteopenia models. Jpn J Pharmacol.

[B49] Gretarsdottir S, Thorleifsson G, Reynisdottir ST, Manolescu A, Jonsdottir S, Jonsdottir T, Gudmundsdottir T, Bjarnadottir SM, Einarsson OB, Gudjonsdottir HM, Hawkins M, Gudmundsson G, Gudmundsdottir H, Andrason H, Gudmundsdottir AS, Sigurdardottir M, Chou TT, Nahmias J, Goss S, Sveinbjornsdottir S, Valdimarsson EM, Jakobsson F, Agnarsson U, Gudnason V, Thorgeirsson G, Fingerle J, Gurney M, Gudbjartsson D, Frigge ML, Kong A, Stefansson K, Gulcher JR (2003). The gene encoding phosphodiesterase 4D confers risk of ischemic stroke. Nat Genet.

[B50] Ghiselli G, Gregg RE, Zech LA, Schaefer EJ, Brewer HBJ (1982). Phenotype study of apolipoprotein E isoforms in hyperlipoproteinaemic patients. Lancet.

[B51] Corder EH, Saunders AM, Strittmatter WJ, Schmechel DE, Gaskell PC, Small GW, Roses AD, Haines JL, Pericak-Vance MA (1993). Gene dose of apolipoprotein E type 4 allele and the risk of Alzheimer's disease in late onset families. Science.

[B52] Peacock M, Turner CH, Econs MJ, Foroud T (2002). Genetics of osteoporosis. Endocr Rev.

[B53] Houslay MD, Adams DR (2003). PDE4 cAMP phosphodiesterases: modular enzymes that orchestrate signalling cross-talk, desensitization and compartmentalization. Biochem J.

[B54] Lohmueller KE, Pearce CL, Pike M, Lander ES, Hirschhorn JN (2003). Meta-analysis of genetic association studies supports a contribution of common variants to susceptibility to common disease. Nat Genet.

[B55] Ioannidis JP, Ntzani EE, Trikalinos TA, Contopoulos-Ioannidis DG (2001). Replication validity of genetic association studies. Nat Genet.

[B56] Trikalinos TA, Ntzani EE, Contopoulos-Ioannidis DG, Ioannidis JP (2004). Establishment of genetic associations for complex diseases is independent of early study findings. Eur J Hum Genet.

